# Left ventricular remodelling patterns in patients with moderate aortic stenosis^[Author-notes jeac018-FM1]^

**DOI:** 10.1093/ehjci/jeac018

**Published:** 2022-02-18

**Authors:** Jan Stassen, See Hooi Ewe, Kensuke Hirasawa, Steele C Butcher, Gurpreet K Singh, Mohammed R Amanullah, Kenny Y K Sin, Zee P Ding, Stephan M Pio, Nicholas W S Chew, Ching Hui Sia, William K F Kong, Kian Keong Poh, David J Cohen, Philippe Généreux, Martin B Leon, Nina Ajmone Marsan, Victoria Delgado, Jeroen J Bax

**Affiliations:** Department of Cardiology, Leiden University Medical Center, Albinusdreef 2, 2300 RC Leiden, The Netherlands; Department of Cardiology, Jessa Hospital, Hasselt, Belgium; Department of Cardiology, National Heart Centre Singapore, Singapore, Singapore; Department of Cardiology, Leiden University Medical Center, Albinusdreef 2, 2300 RC Leiden, The Netherlands; Department of Cardiology, Leiden University Medical Center, Albinusdreef 2, 2300 RC Leiden, The Netherlands; Department of Cardiology, Royal Perth Hospital, Perth, Western Australia, Australia; Department of Cardiology, Leiden University Medical Center, Albinusdreef 2, 2300 RC Leiden, The Netherlands; Department of Cardiology, National Heart Centre Singapore, Singapore, Singapore; Department of Cardiology, National Heart Centre Singapore, Singapore, Singapore; Department of Cardiology, National Heart Centre Singapore, Singapore, Singapore; Department of Cardiology, Leiden University Medical Center, Albinusdreef 2, 2300 RC Leiden, The Netherlands; Department of Cardiology, National University Heart Center Singapore, Singapore, Singapore; Department of Cardiology, National University Heart Center Singapore, Singapore, Singapore; Department of Cardiology, National University Heart Center Singapore, Singapore, Singapore; Department of Cardiology, National University Heart Center Singapore, Singapore, Singapore; Department of Cardiology, Saint Francis Hospital, Roslyn, NY, USA; Department of Cardiology, Cardiovascular Research Foundation, New York, NY, USA; Department of Cardiology, Gagnon Cardiovascular Institute, Morristown Medical Center, Morristown, NJ, USA; Department of Cardiology, Columbia University Irving Medical Center/New York—Presbyterian Hospital, Cardiovascular Research Foundation, New York, NY, USA; Department of Cardiology, Leiden University Medical Center, Albinusdreef 2, 2300 RC Leiden, The Netherlands; Department of Cardiology, Leiden University Medical Center, Albinusdreef 2, 2300 RC Leiden, The Netherlands; Department of Cardiology, Leiden University Medical Center, Albinusdreef 2, 2300 RC Leiden, The Netherlands; Department of Cardiology, Turku Heart Center, University of Turku, Turku University Hospital, Turku, Finland

**Keywords:** moderate aortic stenosis, left ventricular remodelling, mortality, aortic valve replacement

## Abstract

**Aims:**

Moderate aortic stenosis (AS) is associated with an increased risk of adverse events. Because outcomes in patients with AS are ultimately driven by the condition of the left ventricle (LV) and not by the valve, assessment of LV remodelling seems important for risk stratification. This study evaluated the association between different LV remodelling patterns and outcomes in patients with moderate AS.

**Methods and results:**

Patients with moderate AS (aortic valve area 1.0–1.5 cm^2^) were identified and stratified into four groups according to the LV remodelling pattern: normal geometry (NG), concentric remodelling (CR), concentric hypertrophy (CH), or eccentric hypertrophy (EH). Clinical outcomes were defined as all-cause mortality and a composite endpoint of all-cause mortality and aortic valve replacement (AVR). Of 1931 patients with moderate AS (age 73 ± 10 years, 52% men), 344 (18%) had NG, 469 (24%) CR, 698 (36%) CH, and 420 (22%) EH. Patients with CH and EH showed higher 3-year mortality rates (28% and 32%, respectively) when compared with patients with NG (19%) (*P* < 0.001). After multivariable adjustment, CH remained independently associated with mortality (HR 1.258, 95% CI 1.016–1.558; *P* = 0.035), whereas both CH (HR 1.291, 95% CI 1.088–1.532; *P* = 0.003) and EH (HR 1.217, 95% CI 1.008–1.470; *P* = 0.042) were associated with the composite endpoint of death or AVR.

**Conclusion:**

In patients with moderate AS, those who develop CH already have an increased risk of all-cause mortality. Assessment of the LV remodelling patterns may identify patients at higher risk of adverse events, warranting closer surveillance, and possibly earlier intervention.

## Graphical Abstract

**Graphical Abstract:**
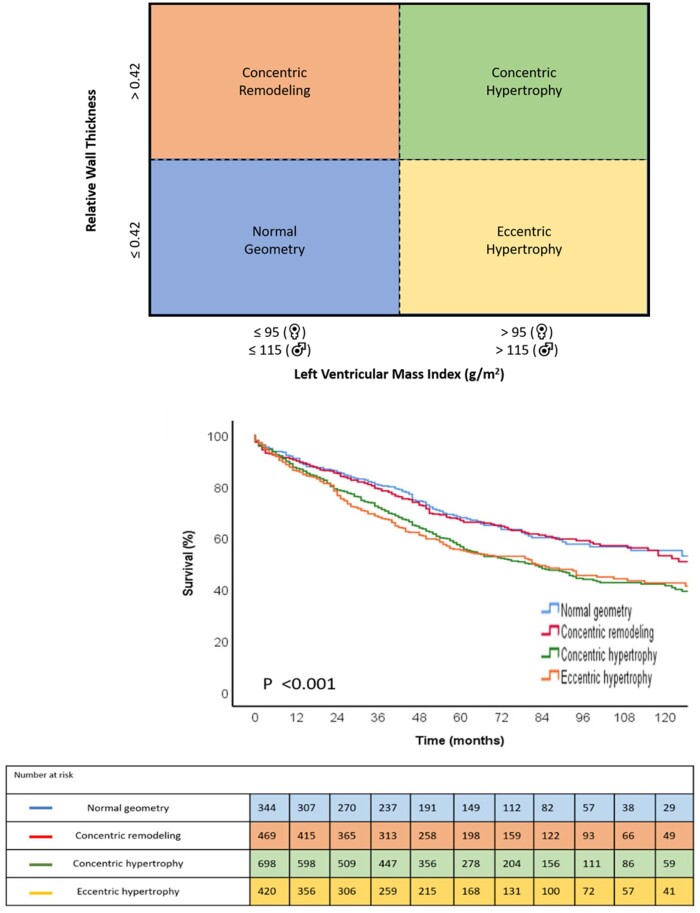
Left ventricular remodeling is already frequently observed in patients with moderate aortic stenosis and is strongly associated with outcomes.

## Introduction

Aortic stenosis (AS) is the most common valvular heart disease in developed countries, affecting 4–5% of patients aged over 65 years.^1,2^ It is well established that symptomatic severe AS is associated with significantly worse survival if left untreated.^3–5^ Recently, less favourable clinical outcomes have also been reported in patients with moderate AS.^6,7^ Hence, identifying patients with moderate AS at higher mortality risk and who may benefit from close surveillance seems crucial. Although risk stratification in AS has traditionally been based on symptoms and left ventricular (LV) ejection fraction (EF),^8,9^ the LV remodelling response to AS-related pressure overload is noted before a reduction in LVEF occurs, with several studies demonstrating that LV structural changes are already apparent despite preserved LVEF.^10,11^ Moreover, adverse LV remodelling is an important predictor of cardiovascular morbidity and mortality in many cardiovascular diseases,^12–14^ including severe AS.^15,16^ Indeed, while LV remodelling is initially a benign response to increased wall stress, it eventually may lead to development of myocardial fibrosis with a progressive deterioration in LV diastolic relaxation and performance, which are both associated with adverse clinical outcomes in severe AS.^10,17–19^ In addition, patients with LV hypertrophy may not always show reverse LV remodelling after aortic valve replacement (AVR), leading to worse postoperative outcomes. These observations underscore the need to identify echocardiographic parameters beyond LVEF to detect the consequences of AS afterload on the LV. Current guidelines recognize four patterns of LV remodelling that could be used to describe the LV response to AS: normal geometry, concentric remodelling, concentric hypertrophy, and eccentric hypertrophy.^20^ The prognostic implications of these four patterns of LV remodelling in patients with moderate AS, however, have not been investigated. In this study, we aimed to investigate the clinical and echocardiographic characteristics of these four LV remodelling patterns and their prognostic implications in patients with moderate AS.

## Methods

### Patient population

From the ongoing registries of patients with moderate aortic valve disease from three academic institutions (Leiden University Medical Center, Leiden, The Netherlands, National University Hospital, Singapore and National Heart Center Singapore, Singapore), patients ≥ 18 years who presented between October 2001 and December 2019 with a first echocardiographic diagnosis of moderate AS were identified. Moderate AS was defined as an aortic valve area (AVA) between 1.0 and 1.5 cm^2^.^8^ The definition of moderate AS based on aortic valve area was used to avoid inclusion of patients with severe, low-flow, low-gradient AS and was based on previous papers.^21,22^ Patients with previous aortic valve surgery, congenital heart disease, bicuspid aortic valve, supra- or subvalvular AS, or dynamic LV outflow tract obstruction were excluded. All patients underwent complete clinical and echocardiographic evaluation at the time of first diagnosis of moderate AS. Patient information was prospectively collected from the departmental cardiology information system and retrospectively analysed. Clinical data included demographic characteristics, cardiovascular risk factors, New York Heart Association (NYHA) functional class, and comorbidities. The study complies with the Declaration of Helsinki and was approved by the institutional review boards of each centre. Due to the retrospective design of the study, the medical ethics committee of each participating centre waived the need for written informed consent.

### Transthoracic echocardiography

**Figure 1:**
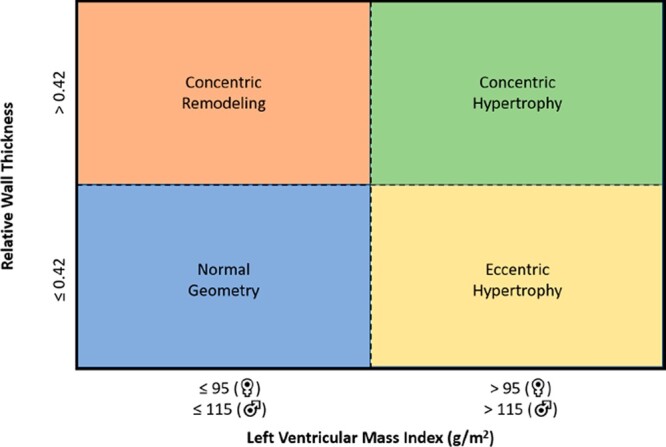
Basic patterns of left ventricular remodelling.

All echocardiographic studies were performed using commercially available ultrasound systems and images were retrospectively analysed in each centre according to current guidelines.^20^ In the parasternal long-axis view, LV dimensions were assessed, and LV mass was calculated using Devereux’s formula and indexed for body surface area (LVMi).^20^ Relative wall thickness (RWT) was calculated with the formula: (2 × posterior wall thickness)/(LV internal diameter at end-diastole).^20^ The echocardiographic variables that define LV geometry (LVMi and RWT) were subsequently used to categorize patients into four patterns of LV remodelling: normal geometry (normal LVMi and RWT ≤0.42), concentric remodelling (normal LVMi and RWT >0.42), concentric hypertrophy (increased LVMi and RWT >0.42), and eccentric hypertrophy (increased LVMi and RWT ≤0.42)^20^ (*Figure 1*). Normal LVMi was defined as LVMi ≤95 g/m^2^ for women and LVMi ≤115 g/m^2^ for men.^20^ LV volumes were assessed and LVEF was calculated according to biplane Simpson’s method.^20^ Left atrial volumes were measured by the biplane method of disks and indexed for body surface area.^20^ From the apical three- or five-chamber views, continuous-wave Doppler recordings were obtained to estimate peak aortic jet velocity.^23^ Mean and peak transvalvular pressure gradients were calculated using the Bernoulli equation.^23^ AVA was calculated using the LV outflow tract diameter and velocity time integrals of the aortic valve and LV outflow tract.^23^ Severity of mitral and tricuspid regurgitation was graded using a multiparametric approach, as recommended by current guidelines.^24^ Pulsed-wave Doppler recordings of the transmitral flow were used to obtain peak early (*E*) and late (*A*) diastolic velocities.^25^ Using tissue Doppler imaging of the mitral annulus on the apical four-chamber view, the *e*′ was measured at both the lateral and septal side, and averaged to calculate the *E*/*e*′ ratio.^25^ The right ventricular systolic pressure was calculated from the peak velocity of the tricuspid regurgitant jet according to the Bernoulli equation, by adding the right atrial pressure determined by the inspiratory collapse and diameter of the inferior vena cava.^20,26^ For the evaluation of right ventricular systolic function, anatomical M-mode was applied to the focused apical four-chamber view of the right ventricle to measure tricuspid annular plane systolic excursion.^26^

### Clinical endpoints

All patients were followed-up for all-cause mortality and the occurrence of AVR (either surgical or transcatheter). The primary outcome was all-cause mortality, which was obtained by review of hospital records linked to the governmental death registry database.

### Statistical analysis

Continuous data are presented as mean ± standard deviation when normally distributed and as median (interquartile range) when not normally distributed. Categorical data are presented as frequencies and percentages. For comparison of continuous variables between groups, the one-way analysis of variance with Bonferroni’s *post hoc* analysis or the Kruskal–Wallis test was used for normally and non-normally distributed variables, respectively. Categorical variables were compared using the Pearson *χ*^2^ test. The inter- and intra-observer variability of LVMi was assessed by calculating the intra-class correlation coefficient on 50 randomly selected patients. The intra-class correlation coefficients for inter- and intra-observer variability were 0.91 [95% confidence interval (CI) 0.88–0.98; *P* < 0.001] and 0.94 (95% CI 0.86–0.97; *P* < 0.001), respectively. Event-free survival curves were generated using the Kaplan–Meier method, and differences between groups were analysed using the log-rank test. Uni- and multivariable analyses of time to events were performed using Cox proportional hazard models with the different patterns of LV remodelling as an independent variable. The following covariables, considered to have a potential prognostic impact, were included: age, sex, diabetes mellitus, arterial hypertension, dyslipidaemia, coronary artery disease, previous myocardial infarction, atrial fibrillation, estimated glomerular filtration rate, NYHA functional class ≥II, LVEF, left atrial volume index, and AVA. The occurrence of surgical or transcatheter AVR was entered as a time-dependent covariate. A separate analysis of time to events using Cox proportional hazard models was performed in the subgroup of patients with preserved LVEF (LVEF ≥50%), and in the subgroup of patients without concomitant significant (≥ moderate) aortic regurgitation. The entry criterium for the multivariable regression analysis was an amount of missing values that did not exceed 10% of the total study population. For both uni- and multivariable analyses, hazard ratios (HRs) with 95% CI were presented. A two-sided *P* value <0.05 was considered statistically significant. Statistical analysis was performed using SPSS for Windows, version 25.0 (IBM, Armonk, NY, USA).

## Results

### Clinical characteristics

A total of 1931 patients (age 73 ± 10 years, 52% men) were included in the study (Supplementary data online, *Figure S1*). Normal geometry was present in 344 (18%) patients, concentric remodelling in 469 (24%) patients, concentric hypertrophy in 698 (36%) patients, and eccentric hypertrophy in 420 (22%) patients. The clinical characteristics of the overall population and according to the different patterns of LV remodelling are shown in *Table 1*. Most patients had arterial hypertension (80%) and dyslipidaemia (74%), while diabetes mellitus was observed in one-third of the patients (34%). A history of coronary artery disease was noted in 865 (45%) patients of whom 362 (19%) had a previous myocardial infarction. Dyspnoea, defined as NYHA functional class ≥II, was observed in 828 (43%) patients and one-third of the patients (34%) used diuretic agents. In per-group analysis, patients with concentric hypertrophy were older than patients with normal geometry and were more likely to be female, had more arterial hypertension, more impaired renal function, and more severe symptoms (NYHA functional class III–IV) compared with patients with concentric remodelling. Patients with eccentric hypertrophy had more coronary artery disease and previous myocardial infarction, more impaired renal function, and more severe symptoms compared to patients with concentric remodelling.

**Table 1:** Clinical characteristics of the total study population and according to different patterns of LV remodelling

Variables	Total population (*n* = 1931)	Normal geometry (*n* = 344)	Concentric remodelling (*n* = 469)	Concentric hypertrophy (*n* = 698)	Eccentric hypertrophy (*n* = 420)	*P*-value
Age (years)	73.2 (± 10.3)	71.9 (± 11.4)	74.3 (± 10.0)^*^	73.9 (± 9.7)^*^	72.1 (± 10.5)^**,***^	<0.001
Male sex (%)	995 (51.5)	200 (58.1)	285 (60.8)	296 (42.2)^*,**^	214 (51.0)^**,***^	<0.001
Arterial hypertension (%)	1546 (80.3)	270 (78.5)	359 (76.7)	588 (84.4)^**^	329 (78.9)	0.006
Dyslipidaemia (%)	1428 (74.3)	254 (73.8)	339 (72.6)	529 (76.1)	306 (73.4)	0.545
DM (%)	661 (34.3)	112 (32.6)	161 (34.4)	252 (36.2)	136 (32.6)	0.561
Current smoker (%)	173 (9.3)	35 (10.6)	31 (7.0)	62 (9.3)	45 (10.9)	0.202
Obesity (BMI ≥30 kg/m^2^) (%)	366 (19.0)	61 (17.7)	87 (18.6)	146 (20.9)	72 (17.1)	0.387
CAD (%)	865 (44.9)	158 (45.9)	184 (39.3)	319 (45.7)	204 (48.8)^**^	0.032
Previous MI (%)	362 (18.8)	65 (19.0)	63 (13.5)	124 (17.8)	110 (26.3)^**,***^	<0.001
Atrial fibrillation (%)	552 (28.6)	86 (25.0)	119 (25.4)	211 (30.2)	136 (32.6)	0.034
Previous stroke (%)	285 (14.8)	48 (14.0)	63 (13.5)	109 (15.6)	65 (15.5)	0.706
COPD (%)	142 (7.4)	25 (7.3)	35 (7.5)	47 (6.7)	35 (8.4)	0.788
NYHA class II–IV (%)	828 (43.4)	135 (39.7)	188 (40.4)	305 (44.3)	200 (48.1)	0.057
NYHA class III–IV (%)	321 (16.8)	49 (14.4)	57 (12.3)	126 (18.3)^**^	89 (21.4)^**^	0.001
Angina (%)	162 (8.5)	24 (7.0)	38 (8.2)	50 (7.3)	50 (12.0)^***^	0.030
Syncope (%)	30 (1.6)	7 (2.1)	9 (1.9)	8 (1.2)	6 (1.4)	0.641
Beta-blocker (%)	949 (49.4)	165 (48.0)	217 (46.5)	343 (49.6)	224 (53.6)	0.184
ACEi or ARB (%)	957 (49.8)	165 (48.0)	215 (46.0)	365 (52.8)	212 (50.7)	0.123
MRA (%)	109 (5.7)	19 (5.6)	18 (3.9)	29 (4.2)	43 (10.3)^**,***^	<0.001
Diuretic (%)	661 (34.4)	101 (29.4)	137 (29.3)	236 (34.2)	187 (44.7)^*,**,***^	<0.001
CCB (%)	757 (39.4)	128 (37.2)	164 (35.1)	315 (45.6)^**^	150 (35.9)^***^	0.001
Statin (%)	1340 (69.8)	250 (72.7)	323 (69.2)	484 (70.0)	283 (67.7)	0.507
Aspirin (%)	904 (47.1)	160 (46.5)	222 (47.5)	324 (46.9)	198 (47.4)	0.991
Oral anticoagulation (%)	387 (20.2)	62 (18.0)	88 (18.8)	131 (19.0)	106 (25.4)	0.028
eGFR (mL/min/1.73 m^2^)	67 (44–87)	74 (53–93)	71.4 (53–90)	61 (35–83)^*,**^	66 (40–86)^*,**^	<0.001
Haemoglobin (g/dL)	12.5 (11.0–13.7)	12.5 (10.9–13.7)	13.0 (11.6–14.1)^*^	12.1 (10.6–13.4)^**^	12.4 (11.0–13.6)^**^	<0.001

Values are presented as mean ± SD, median (IQR), or *n* (%).

ACEi, angiotensin-converting enzyme inhibitor; ARB, angiotensin receptor blocker; BMI, body mass index; CAD, coronary artery disease; CCB, calcium channel blocker; COPD, chronic obstructive pulmonary disease; DM, diabetes mellitus; eGFR, estimated glomerular filtration rate; MI, myocardial infarction; MRA, mineralocorticoid receptor antagonist; NYHA, New York Heart Association.

* *P* < 0.05 vs. Group I.

** *P* < 0.05 vs. Group II.

*** *P* < 0.05 vs. Group III.

### Echocardiographic variables

*Table 2* summarizes the echocardiographic data of the study population. Mean LVEF was 58 ± 13% with 372 (19%) patients having LVEF <50%. Mean AVA was 1.22 ± 0.15 cm^2^, mean aortic gradient 23 ± 9 mmHg, and peak aortic jet velocity 3.0 ± 0.6 m/s. In the per-group analysis, patients with concentric hypertrophy had more severe LV diastolic dysfunction (with higher *E*/*e*′ and larger left atrial volumes), whereas patients with eccentric hypertrophy had larger LV dimensions, lower LVEF, more severe LV diastolic dysfunction, and higher systolic pulmonary artery pressures compared to patients with normal geometry. In addition, patients with eccentric hypertrophy had a higher prevalence of concomitant significant aortic and mitral regurgitation. Interestingly, patients with concentric hypertrophy had higher peak aortic jet velocities and mean aortic gradients compared to patients with normal geometry.

**Table 2:** Echocardiographic characteristics of the total study population and according to different patterns of remodelling

Variables	Total population (*n* = 1931)	Normal geometry (*n* = 344)	Concentric remodelling (*n* = 469)	Concentric hypertrophy (*n* = 698)	Eccentric hypertrophy (*n* = 420)	*P*-value
LV EDD (mm)	48.0 (± 7.2)	48.1 (± 5.0)	41.8 (± 4.6)^*^	47.3 (± 5.5)^**^	55.7 (± 6.3)^*,**,***^	<0.001
LV ESV (mL)	38 (28–53)	40 (32–51)	30 (22–38)^*^	38 (28–50)^**^	58 (41–85)^*,**,***^	<0.001
LV EDV (mL)	98 (79–124)	99 (85–118)	80 (66–97)^*^	97 (79–118)^**^	131 (107–156)^*,**,***^	<0.001
LVEF (%)	58.2 (± 12.6)	58.0 (± 11.7)	62.3 (± 9.4)^*^	59.6 (± 11.2)^**^	51.4 (± 15.7)^*^^,**,***^	<0.001
LVMi (g/m^2^)	115.9 (± 34.8)	85.9 (± 15.0)	88.3 (± 14.6)	135.7 (± 29.3)^*,**^	138.3 (± 31.3)^*,**^	<0.001
LAVi (mL/m^2^)	37 (29–47)	33 (28–43)	32 (26–41)	39 (31–49)^*,**^	42 (34–53)^*,**,***^	<0.001
*E*/*e*′	14.5 (10.9–20.0)	13.1 (9.6–17.5)	13.0 (9.8–18.0)	15.2 (11.6–20.8)^*,**^	16.6 (12.3–21.8)^*,**^	<0.001
Moderate or severe MR (%)	181 (9.4)	34 (9.9)	14 (3.0)^*^	58 (8.3)^**^	75 (17.9)^*,**,^^***^	<0.001
Stroke volume index (mL/m^2^)	48.2 (± 13.1)	46.4 (± 12.8)	46.6 (± 11.3)	50.2 (± 12.0)^*,**^	48.3 (± 16.0)	<0.001
Peak aortic velocity (m/s)	3.0 (± 0.6)	2.9 (± 0.6)	3.1 (± 0.6)^*^	3.1 (± 0.6)^*^	3.0 (± 0.6)^**,***^	<0.001
Aortic mean pressure gradient (mmHg)	22.7 (± 8.6)	21.3 (± 8.1)	23.0 (± 8.5)^*^	23.8 (± 8.6)^*^	21.9 (± 9.0)^***^	<0.001
Aortic valve area (cm)	1.22 (± 0.15)	1.23 (± 0.15)	1.23 (± 0.15)	1.21 (± 0.15)	1.22 (± 0.15)	0.091
Moderate or severe AR (%)	210 (10.9)	31 (9.0)	41 (8.7)	71 (10.2)	67 (16.0)^*,**,^^***^	0.002
TAPSE (mm)	21 (19–24)	22 (19–25)	22 (19–25)	21 (18–24)	20 (18–24)^*,**^	0.005
PASP (mmHg)	33 (27–42)	33 (26–41)	32 (25–38)	33 (28–42)^**^	36 (28–46)^*,**^	<0.001
Moderate or severe TR (%)	313 (16.3)	57 (16.7)	69 (14.8)	101 (14.6)	86 (20.6)	0.050

Values are presented as mean ± SD, median (IQR), or *n* (%).

AR, aortic regurgitation; EDD, end-diastolic diameter; EDV, end-diastolic volume; ESV, end-systolic volume; EF, ejection fraction; ESD, end-systolic diameter; LAVI, left atrial volume index; LV, left ventricular; LVMi, left ventricular mass index; MR, mitral regurgitation; PASP, pulmonary artery systolic pressure; TAPSE, tricuspid annular plane systolic excursion; TR, tricuspid regurgitation.

* *P* < 0.05 vs. Group I.

** *P* < 0.05 vs. Group II.

*** *P* < 0.05 vs. Group III.

### Prognostic impact of LV remodelling patterns

During a median follow-up of 51 (25–83) months, 833 (43%) patients died. The cumulative 1-, 3-, and 5-year survival rates were 88%, 74%, and 61%, respectively. For the composite endpoint of death or AVR, 1286 (67%) underwent AVR (*n* = 613; 48%) or died (*n* = 673; 52%) during a median follow-up of 35 (14–60) months. Of the 613 patients who underwent AVR, 250 (41%) patients underwent transcatheter AVR, and 363 (59%) patients underwent surgical AVR.

**Figure 2:**
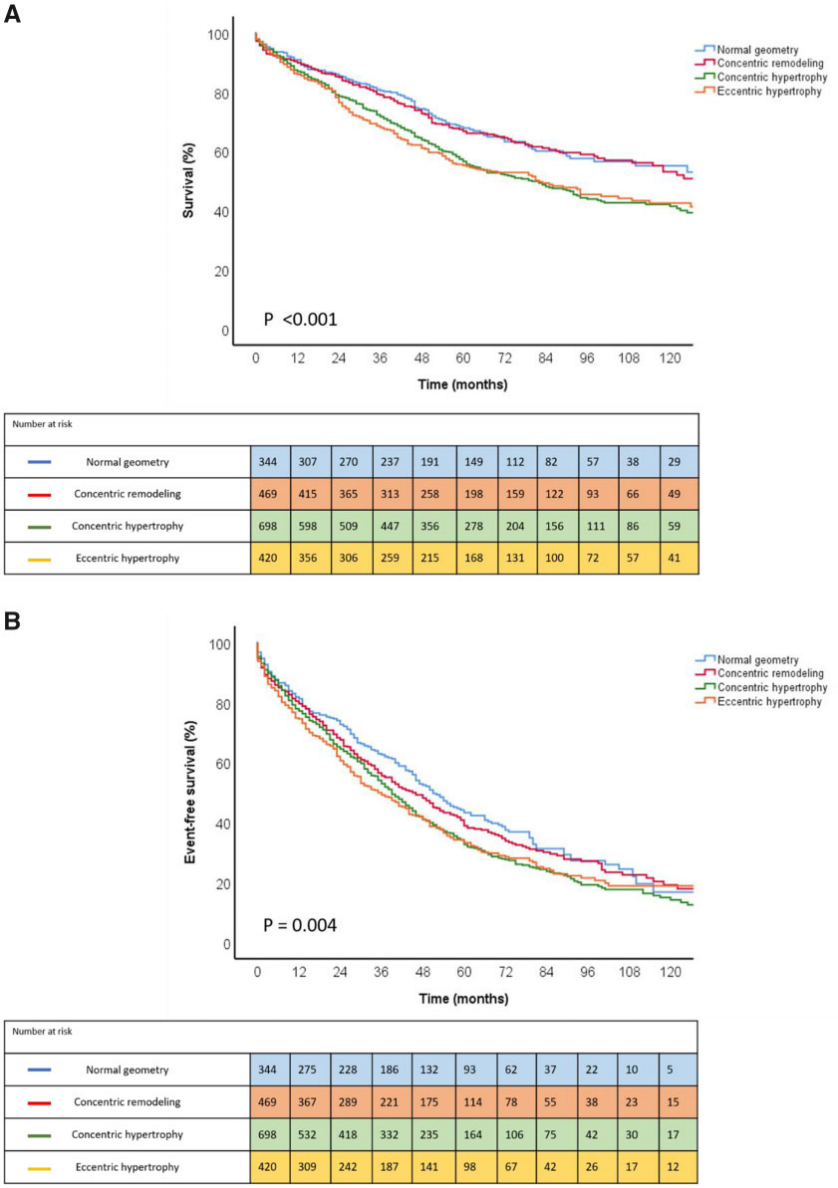
Kaplan–Meier curves for all-cause mortality (*A*) and the composite endpoint of death and AVR (*B*) according to different patterns of LV remodelling. AVR, aortic valve replacement; LV, left ventricular.

The Kaplan–Meier survival curves demonstrated significantly lower survival rates at 1-, 3-, and 5-year follow-up in patients with concentric hypertrophy (87%, 72%, and 57%, respectively) and eccentric hypertrophy (86%, 68%, and 55%, respectively), when compared with patients with normal geometry (91%, 81%, and 68%, respectively) (*P* < 0.001; *Figure 2A*). Similarly, patients with concentric hypertrophy and eccentric hypertrophy showed significantly lower 1-, 3-, and 5-year event-free survival rates for the composite endpoint of death and AVR (77%, 53%, and 33%, and 75%, 49%, and 33%, respectively) when compared with patients with normal geometry (81%, 63%, and 43%, respectively) (*P* = 0.004; *Figure 2B*). Similar relationships were observed in patients without significant aortic regurgitation (Supplementary data online, *Figure S2*). In the subgroup of patients with preserved LVEF (LVEF ≥50%), patients with concentric hypertrophy, but not with eccentric hypertrophy, had significantly worse survival outcomes (*P* = 0.001), as well as event-free survival outcomes for the composite endpoint of death and AVR (*P* = 0.009), compared to patients with normal geometry (Supplementary data online, *Figure S3*).

The multivariable Cox model is shown in *Table 3*. All-cause mortality was adjusted for AVR as a time-dependent covariate. On multivariable analysis, only concentric hypertrophy was independently associated with all-cause mortality (HR 1.258, 95% CI 1.016–1.558; *P* = 0.035), whereas both concentric hypertrophy (HR 1.291, 95% CI 1.088–1.532; *P* = 0.003) and eccentric hypertrophy (HR 1.217, 95% CI 1.008–1.470; *P* = 0.042) were independently associated with the composite endpoint of death and AVR. Similar associations with outcomes were observed for patients with LVEF ≥50% (Supplementary data online, *Table S1*), while in patients without significant aortic regurgitation, only concentric hypertrophy was significantly associated with all-cause mortality and the composite endpoint of death and AVR (Supplementary data online, *Table S2*). When considering LV remodelling according to LVMi as a continuous variable (instead of the 4 remodelling patterns), LVMi was independently associated with all-cause mortality (HR 1.003, 95% CI 1.001–1.005; *P* = 0.001), as well as the composite endpoint of death and AVR (HR 1.002, 95% CI 1.001–1.004; *P* = 0.004).

**Table 3:** Univariable and multivariable Cox regression analysis for all-cause mortality and the composite endpoint of death and AVR

	All-cause mortality		AVR or all-cause mortality	
	HR (95% CI)	*P* value	HR (95% CI)	*P* value
	Univariable analysis		Univariable analysis	
Normal geometry	*Reference group*		*Reference group*	
Concentric remodelling	0.976 (0.773–1.233)	0.840	1.103 (0.922–1.319)	0.282
Concentric hypertrophy	1.410 (1.147–1.734)	0.001	1.285 (1.091–1.513)	0.003
Eccentric hypertrophy	1.435 (1.148–1.793)	0.002	1.300 (1.087–1.555)	0.004
	**Multivariable analysis** ^a^		**Multivariable analysis** ^b^	
Normal geometry	*Reference group*		*Reference group*	
Concentric remodelling	0.973 (0.763–1.240)	0.825	1.111 (0.921–1.342)	0.272
Concentric hypertrophy	1.258 (1.016–1.558)	0.035	1.291 (1.088–1.532)	0.003
Eccentric hypertrophy	1.244 (0.987–1.568)	0.065	1.217 (1.008–1.470)	0.042

AVR, aortic valve replacement; CI, confidence interval; HR, hazard ratio.

a Adjusted for age, sex, arterial hypertension, diabetes mellitus, hyperlipidaemia, coronary artery disease, previous myocardial infarction, atrial fibrillation, estimated glomerular filtration rate, NYHA class ≥2, left ventricular ejection fraction, left atrial volume index, aortic valve area, and SAVR/TAVR as a time-dependent covariate.

b Adjusted for age, sex, arterial hypertension, diabetes mellitus, hyperlipidaemia, coronary artery disease, previous myocardial infarction, atrial fibrillation, estimated glomerular filtration rate, NYHA class ≥2, left ventricular ejection fraction, left atrial volume index, and aortic valve area.

Interestingly, AVR was significantly associated with a lower mortality rate (HR 0.642, 95% CI 0.528–0.782; *P* < 0.001). There was no interaction between the type of intervention (surgical vs. transcatheter AVR) and remodelling patterns with outcomes (*P* = 0.248).

## Discussion

The main findings of this study with data obtained from a large registry of patients with moderate AS can be summarized as follows: (i) LV remodelling varies significantly in patients with moderate AS, and (ii) LV concentric hypertrophy is independently associated with all-cause mortality after adjusting for various clinical and echocardiographic variables.

### LV remodelling in moderate AS

Although LV remodelling is considered a compensatory mechanism to reduce wall stress and maintain LVEF in AS, it eventually becomes maladaptive, leading to a progressive impairment in LV diastolic relaxation and LV performance which are both associated with worse outcomes in patients with AS.^17–19,27^ Indeed, as the LV hypertrophies, myocardial oxygen demand increases while coronary flow reserve decreases due to concomitant microvascular dysfunction, low coronary perfusion pressure, increased extravascular compressive forces, and reduced diastolic perfusion time.^28,29^ This disbalance between oxygen demand and delivery eventually causes ischaemia and leads to LV myocardial fibrosis, which is known to be a major determinant of clinical progression from compensated LV hypertrophy to heart failure and is independently associated with mortality in patients with severe AS.^10,30–32^ Previous studies have already demonstrated the association between LV mass and LV myocardial fibrosis detected by CMR,^32^ which could explain why a higher LV mass detected by echocardiography is associated with an increased risk of heart failure in AS^33^ and is associated with worse outcomes in this study.

Although AVR relieves the imposed valvular afterload on the LV, LV diastolic dysfunction may persist due to the underlying presence of irreversible LV myocardial fibrosis. Therefore, end-diastolic LV pressure remains high after AVR and this could generate a vicious cycle of reduced coronary artery perfusion with further progression of LV mid-wall fibrosis. These findings might explain why maladaptive LV remodelling does not necessarily reverse after AVR.^10,27,34–36^ In addition, if no timely intervention is performed, the compensatory mechanisms to overcome the increase in wall stress could eventually become insufficient, leading to a progressive reduction in LVEF. Inverse correlations between LVEF and LV myocardial fibrosis have been demonstrated by Hein *et al.*,^30^ suggesting that LV myocardial fibrosis significantly contributes to the progression of LV systolic dysfunction. These observations demonstrate that the LV response to valvular afterload occurs through a continuous spectrum of LV remodelling and emphasize the importance of a timely intervention at the level of the aortic valve, maybe even when AS severity is still moderate.

### Prognostic value of LV remodelling patterns in moderate AS

Although the traditional focus of AS assessment has been on the valve, it is increasingly acknowledged that AS is not only a disease of the aortic valve but also of the LV. Current guidelines strongly recommend AVR for patients with severe AS who are either symptomatic or develop LV systolic dysfunction, demonstrating that the decision to intervene is ultimately driven by the condition of the LV myocardium and not by the valve. This study shows that LV remodelling in patients with moderate AS already varies considerably and that different patterns of LV remodelling have a different impact on prognosis. Interestingly, only concentric hypertrophy remained independently associated with all-cause mortality after adjusting for other clinical and echocardiographic variables. The observation that concentric hypertrophy is associated with outcomes in patients with a pressure overloaded LV has been previously demonstrated. Koren *et al.*^37^ showed that patients with essential arterial hypertension and concentric hypertrophy had a two-fold increase in cardiovascular deaths compared to patients with eccentric hypertrophy. Similarly, in 436 hypertensive patients, Muiesan *et al*.^13^ demonstrated that cardiovascular morbidity and mortality were significantly higher in patients with concentric hypertrophy compared to patients with eccentric hypertrophy. In addition, Capoulade *et al.*^15^ demonstrated that LV concentric hypertrophy was independently associated with an increased risk of mortality in patients with severe AS. Our data expand on these results by demonstrating a strong, independent link between concentric LV hypertrophy and outcomes in a large population of patients with moderate AS.

In this study, patients with concentric hypertrophy had higher peak aortic jet velocities and mean aortic pressure gradients with a non-significant trend to a smaller AVA compared to patients with a normal geometry. These observations suggest that AS severity might need to be considered as a continuous variable, with each incremental increase in AS severity imposing an additional pressure load on the LV, thereby facilitating LV remodelling. In addition, it is important to notify that LV remodelling is not only a manifestation of the underlying AS, but rather an expression of the complex interaction between the LV myocardium, valvular afterload, and arterial afterload. In the current study, patients with moderate AS had a high prevalence of concomitant cardiovascular comorbidities, such as diabetes mellitus, arterial hypertension, coronary artery disease, and significant concomitant aortic and mitral regurgitation, which are all known to have an impact on LV remodelling (and the development of LV fibrosis) as well.^13,37,38^ Nonetheless, it has been demonstrated that in patients with a combination of moderate AS and reduced systemic arterial compliance, global LV afterload is equivalent, and has similar adverse effects on LV remodelling, compared to patients with isolated severe AS.^39^ Any significant decrease in LV afterload may therefore contribute to the improvement of LV remodelling and future trials are needed to investigate if treating moderate AS in well-selected patients with a markedly increased valvulo-arterial impedance could reverse LV remodelling and improve outcomes.

### Clinical implications

Current guidelines recommend AVR for patients with severe AS who are symptomatic or in whom LV systolic dysfunction develops in the absence of symptoms.^8,9^ However, as shown in this study, the clinical and echocardiographic presentation of patients with moderate AS already varies considerably with a large proportion of patients showing marked LV remodelling. Because different patterns of LV remodelling are closely related to prognosis, characterizing these patterns in patients with moderate AS is important in daily clinical practice. Whether these data should expand current indications for AVR before progression to severe AS and outweigh the risk of intervention remains uncertain, especially considering the high prevalence of concomitant cardiovascular comorbidities. Nonetheless, future trials investigating the risk-to-benefit ratio of AVR in patients with moderate AS at increased risk appear warranted. The PROGRESS Trial (A Prospective, Randomized, Controlled Trial to Assess the Management of Moderate Aortic Stenosis by Clinical Surveillance or Transcatheter Aortic Valve Replacement) (NCT 04889872) is currently recruiting patients to explore the hypothesis that transcatheter AVR could improve outcomes in patients with moderate AS.

### Limitations

The limitations of this study are inherent in its retrospective design. Evaluation of LVMi according to Devereux’s formula is based on geometrical assumptions and may not always be applicable (e.g. in case of major LV distortions, such as myocardial infarction). Echocardiographic measurements were not performed in an independent echocardiographic laboratory. A time span of 18 years was used for inclusion of patients to acquire the large cohort as presented. Specific indications for surgery during follow-up were not recorded in the present database. However, follow-up was performed by cardiologists with expertise in valvular heart disease and surgical decisions were made in accordance to current practice guidelines. Patients had a high prevalence of concomitant cardiovascular comorbidities, which could have an impact on LV remodelling. In addition, measuring the valvulo-arterial impedance could provide additional information, but the retrospective design precluded obtaining the necessary data for its calculation. All-cause mortality was chosen as the primary endpoint because the exact cause of death was not systematically recorded.

## Conclusions

Different patterns of LV remodelling are observed in patients with moderate AS with LV concentric hypertrophy being independently associated with all-cause mortality at long-term follow-up. Risk stratification according to the different patterns of LV remodelling may help to identify patients with moderate AS who are at increased risk of adverse events and may benefit from close monitoring and follow-up.

## Supplementary data

Supplementary data are available at *European Heart Journal - Cardiovascular Imaging* online.

## Funding

J.S. received funding from the European Society of Cardiology (ESC Training Grant App000064741). S.C.B. received funding from the European Society of Cardiology (ESC Research Grant App000080404). S.M.P. received funding from the European Society of Cardiology (ESC Training Grant T-2018-17405).

## Supplementary Material

jeac018_Supplementary_Data

## Data Availability

The data underlying this article will be shared on reasonable request to the corresponding author.
