# Supplementary material for: Left ventricular remodelling patterns in patients with moderate aortic stenosis
Source: Eur Heart J Cardiovasc Imaging. 2022 Feb 18;23(10):1326–35. doi: 10.1093/ehjci/jeac018 (PMC9463993; doi:10.1093/ehjci/jeac018)
Supplement: jeac018_Supplementary_Data [file jeac018_supplementary_data.zip › Table_S1.docx]

**Table S1 – Uni -and multivariable Cox regression analysis for all-cause mortality and the cmposite endpoint of death and AVR in patients with moderate AS and LVEF ≥50%.**

|  | All-cause mortality | | AVR or all-cause mortality | |
| --- | --- | --- | --- | --- |
|  | **HR (95% CI)** | **P value** | **HR (95% CI)** | **P value** |
|  | **Univariable analysis** | | **Univariable analysis** | |
| Normal geometry | *Reference group* |  | *Reference group* |  |
| Concentric remodeling | 1.129 (0.870 – 1.466) | 0.361 | 1.246 (1.022 – 1.518) | 0.029 |
| Concentric hypertrophy | 1.511 (1.190 – 1.920) | 0.001 | 1.372 (1.140 – 1.652) | 0.001 |
| Eccentric hypertrophy | 1.277 (0.963 – 1.694) | 0.090 | 1.210 (0.970 – 1.509) | 0.091 |
|  | **Multivariable analysis ^*^** | | **Multivariable analysis ^**^** | |
| Normal geometry | *Reference group* |  | *Reference group* |  |
| Concentric remodeling | 0.999 (0.762 – 1.310) | 0.994 | 1.174 (0.953 – 1.446) | 0.132 |
| Concentric hypertrophy | 1.293 (1.009 – 1.657) | 0.042 | 1.389 (1.143 – 1.688) | 0.001 |
| Eccentric hypertrophy | 1.246 (0.933 – 1.663) | 0.137 | 1.311 (1.042 – 1.649) | 0.021 |

* Adjusted for the same variables used in Table 3

** Adjusted for the same variables used in Table 3

AS = aortic stenosis; AVR = aortic valve replacement; CI = confidence interval; HR = hazard ratio; LVEF = left ventricular ejection fraction.
