# Supplementary material for: Left ventricular remodelling patterns in patients with moderate aortic stenosis
Source: Eur Heart J Cardiovasc Imaging. 2022 Feb 18;23(10):1326–35. doi: 10.1093/ehjci/jeac018 (PMC9463993; doi:10.1093/ehjci/jeac018)
Supplement: jeac018_Supplementary_Data [file jeac018_supplementary_data.zip › Table_S2.docx]

**Table S2 – Uni -and multivariable Cox regression analysis for all-cause mortality and the composite endpoint of death and AVR in patients with moderate AS but without significant (i.e. ≥ moderate) AR.**

|  | All-cause mortality | | AVR or all-cause mortality | |
| --- | --- | --- | --- | --- |
|  | **HR (95% CI)** | **P value** | **HR (95% CI)** | **P value** |
|  | **Univariable analysis** | | **Univariable analysis** | |
| Normal geometry | *Reference group* |  | *Reference group* |  |
| Concentric remodeling | 0.991 (0.778 – 1.261) | 0.938 | 1.116 (0.9266 – 1.346) | 0.249 |
| Concentric hypertrophy | 1.454 (1.173 – 1.802) | 0.001 | 1.326 (1.117 – 1.573) | 0.001 |
| Eccentric hypertrophy | 1.475 (1.165 – 1.866) | 0.001 | 1.327 (1.096 – 1.607) | 0.004 |
|  | **Multivariable analysis ^*^** | | **Multivariable analysis ^**^** | |
| Normal geometry | *Reference group* |  | *Reference group* |  |
| Concentric remodeling | 0.964 (0.749 – 1.241) | 0.775 | 1.116 (0.916 – 1.360) | 0.276 |
| Concentric hypertrophy | 1.269 (1.016 – 1.584) | 0.036 | 1.330 (1.111 – 1.592) | 0.002 |
| Eccentric hypertrophy | 1.199 (0.940 – 1.531) | 0.144 | 1.203 (0.983 – 1.473) | 0.072 |

* Adjusted for the same variables used in Table 3

** Adjusted for the same variables used in Table 3

AR = aortic regurgitation; AS = aortic stenosis; AVR = aortic valve replacement; CI = confidence interval; HR = hazard ratio.
